# Syringic acid attenuates sodium arsenite-induced hepatotoxicity and diabetes in mice via suppression of oxidative stress/inflammation/apoptosis pathways

**DOI:** 10.22038/ajp.2025.25519

**Published:** 2025

**Authors:** Ali Vadizadeh, Mahdieh Sadat Badiee, Ehsan Saburi, Fereshtesadat Fakhredini, Hadi Kalantar, Sirous Rafiei Asl, Mohammad Javad Khodayar

**Affiliations:** 1 *Student Research Committee, Ahvaz Jundishapur University of Medical Sciences, Ahvaz, Iran*; 2 *Department of Toxicology, Faculty of Pharmacy, Ahvaz Jundishapur University of Medical Sciences, Ahvaz, Iran*; 3 *Department of Medical Genetics and Molecular Medicine, School of Medicine, Mashhad University of Medical Sciences, Mashhad, Iran*; 4 *Medical Genetics Research Center, Mashhad University of Medical Sciences, Mashhad, Iran*; 5 *Cellular and Molecular Research Center, Medical Basic Sciences Research Institute, Ahvaz Jundishapur University of Medical Sciences, Ahvaz, Iran*; 6 *Toxicology Research Center, Medical Basic Sciences Research Institute, Ahvaz Jundishapur University of Medical Sciences, Ahvaz, Iran*; 7 *Cancer, Environmental and Petroleum Pollutants Research Center, Ahvaz Jundishapur University of Medical Sciences, Ahvaz, Iran*

**Keywords:** Sodium arsenite, Hepatotoxicity, Diabetes, Syringic acid, Anti-inflammatory, Antiapoptotic

## Abstract

**Objective:**

Chronic exposure to arsenic increases the risk of type 2 diabetes. Syringic acid (SYRA) has anti-inflammatory and antidiabetic properties. The aim of this study was to investigate the effects of SYRA on sodium arsenite-induced hepatotoxicity and diabetes in mice.

**Materials and Methods:**

Thirty male mice were divided into five groups (n=6), include control, SYRA (25 mg/kg, last week), sodium arsenite (As, 3 mg/kg for 30 days), and therapeutic groups of SYRA (10 and 25 mg/kg, last week). The mice were fasted overnight and fasting blood sugar (FBS), and glucose tolerance test (GTT) were performed. Then the mice were anesthetized, and samples of blood and liver tissue were collected for measurement of alanine aminotransferase (ALT), aspartate aminotransferase (AST), alkaline phosphatase (ALP), superoxide dismutase (SOD), catalase (CAT), glutathione peroxidase (GPx), thiobarbituric acid reactive substances (TBARS), total thiol, nitric oxide (NO), tumor necrosis factor-alpha (TNF-α), and caspase-3 protein expression.

**Results:**

SYRA before As, reduced levels of liver enzymes, FBS, GTT, NO, TNF-α, and TBARS, and elevated levels of total thiol, CAT, SOD, GPx and caspase-3 expression compared to As group in mice.

**Conclusion:**

SYRA can be suggested as a treatment option against the hepatotoxic and diabetogenic effects of As.

## Introduction

Arsenic is an environmental pollutant in organic and inorganic forms, with the trivalent inorganic form being the most toxic (Lu et al., 2014). Arsenic is present in soil, water, air, and food (Mazumder and pharmacology, 2005). Consumption of drinking water contaminated with arsenic is a significant public health issue in numerous countries, including Bangladesh and India (Rouwane et al., 2016). Arsenic is well-known for its carcinogenic, mutagenic, and genotoxic effects (Manna et al., 2007). This highly toxic metal can disrupt insulin secretion by generating free radicals and forming covalent bonds with disulfide bridges in insulin molecules, leading to physiological dysfunction (Singh et al., 2020). Epidemiological studies have shown an association between chronic arsenic exposure and an increased risk of cardiovascular disorders, diabetes, and cancer (Khosravi-Darani et al., 2022). Arsenic exposure contributes to the development of type 2 diabetes through various mechanisms, such as phosphate substitution with arsenate in the mitochondrial electron transport chain, binding to sulfhydryl groups, induction of oxidative stress, impaired insulin secretion and signaling, and impaired glucose homeostasis in peripheral tissues (Castriota et al., 2020; Tseng and pharmacology, 2004). Flavonoids and phenolic acids, as essential secondary metabolites in plants, are known for their anti-inflammatory and antioxidant properties that protect cells against lipid peroxidation, cellular damage, and oxidative stress (Amirmostofian et al., 2023; Güven et al., 2015).

 Syringic acid (SYRA) is a phenolic compound abundantly found in various plants and foods such as beet leaves, olives, walnuts, dates, pumpkins, and grains (Kim et al., 2006; Neveu et al., 2010; Taghizadeh et al., 2018). It is antioxidant, antiproliferative (Hirota et al., 2000), hepatoprotective (Itoh et al., 2009), antiendotoxic (Yunhai et al., 2003), anticancer (Kampa et al., 2004), and neuroprotective (Tokmak et al., 2015). SYRA inhibits lipoprotein oxidation, scavenges free radicals, and reduces malondialdehyde production (Morton et al., 2000). The methoxy groups of SYRA are responsible for scavenging free radical (Srinivasulu et al., 2018). Previous studies have shown that SYRA ameliorates complications of hepatitis, nephropathy, and neuropathy in diabetic mice, indicating its potential as an anti-inflammatory and antidiabetic agent (Sabahi et al., 2021). Given the multiple mechanisms by which arsenic induces diabetes, particularly oxidative stress, and considering the antioxidant and antidiabetic properties of SYRA, we hypothesized that SYRA may protect mice against sodium arsenite-induced hepatic damage and diabetic conditions. Therefore, the present study aimed to investigate the protective effects of SYRA on arsenic-induced hepatotoxicity and diabetes in mice, focusing on the suppression of oxidative stress, inflammation, and apoptosis pathways.

## Materials and Methods

### Chemicals and reagents

Syringic acid (purity ≥99%, CAS: 530-57-4) and sodium arsenite (purity ≥90%, CAS: 7784-46-5) were bought from Sigma-Aldrich (USA). Kits for determining the activity of alanine aminotransferase (ALT), aspartate aminotransferase (AST), and alkaline phosphatase (ALP) were purchased from Pars Azmoon (Tehran, Iran). The tumor necrosis factor-alpha (TNF-α) ELISA kit was obtained from Karmania Pars Gene (Kerman, Iran). Kits for measuring nitric oxide (NO), superoxide dismutase (SOD) and glutathione peroxidase (GPx) were acquired from ZellBio (Germany). 

### Animals

Thirty male NMRI mice (6–8 weeks old, weighing 25±2 g) were housed under standard conditions (12 hr light/12 hr dark cycle, 23±2°C) with unlimited access to food and water. The ethical guidelines for the use and care of laboratory animals were based on the protocol of Ahvaz Jundishapur University of Medical Sciences (ethical code: IR.AJUMS.ABHC.REC.1401.051).

### Experimental design

The animals were divided into five groups (n=6), include control, SYRA (25 mg/kg, gavage), sodium arsenite (As, 3 mg/kg for 30 days, gavage), and therapeutic groups of SYRA (10 and 25 mg/kg). SYRA was administered last week of the study from days 24 to 30. The doses of As and SYRA were selected based on previous studies (Fang et al., 2019; Itoh et al., 2009; Singh et al., 2020). After 30 days of treatment and one night of fasting, fasting blood sugar (FBS) and glucose tolerance test (GTT) were measured using a glucometer (Accu-Chek, Roche Diagnostics, Switzerland). For GTT, glucose (2 g/kg) was injected intraperitoneally into the mice and blood glucose levels were measured at 15, 30 and 60 minutes after injection. After induction of anesthesia with ketamine (90 mg/kg) and xylazine (10 mg/kg), cardiac blood was collected and after centrifugation and serum separation, stored at –20 °C for subsequent evaluations. Liver tissue was removed, washed with normal saline and divided into two parts. One part was fixed in 10% formalin for histopathological examination. The other part was stored at –70°C for biochemical assays.

### Measurement of serum and tissue factors

 Serum ALT, AST, and ALP levels were determined using an autoanalyzer system (BT3000, Biotecnica Instruments, Rome, Italy).

 Liver homogenates were prepared in phosphate buffer, and the supernatant was used to measure TBARS, total thiol, CAT, SOD, GPx, NO and TNF-α levels.

 Protein concentration was determined using the Bradford method (Bistgani et al., 2017).

 Ellman's reagent was used to determine the total thiol content by forming yellow 5-thio-2-nitrobenzoic acid (TNB) (Jafarian et al., 2013).

 TBARS concentration was determined following the protocol outlined by Kei (Kei, 1978).

 CAT activity was determined using the method of Shangari (Shangari and O'Brien, 2006).

 SOD and GPx activities as well as NO levels were measured using commercial kits (ZellBio, Germany). 

### Western blotting

Homogenized liver tissue was centrifuged, and protein concentration was determined and transferred to sodium dodecyl sulfate–polyacrylamide gel electrophoresis (SDS-PAGE), and then polyvinylidene difluoride (PVDF) membranes. Caspase-3 protein was detected by incubating the membranes with a specific primary antibody (Proteintech Group, Inc., Cat No. 19677-1-AP) overnight at 4°C. The next day, it was incubated with a secondary antibody. Finally, the obtained protein band was analyzed using Image J software (NIH, Bethesda, MD, USA). GAPDH (Cat No. 5174, Cell Signaling Technology, USA) enzyme was used as a loading control in Western blotting.

### Histopathological examination

Formalin-fixed liver tissues were processed, embedded in paraffin, and sectioned at five-micrometer thickness. The sections were stained with hematoxylin and eosin (H&E) and examined under a light microscope (Olympus BX51, Tokyo, Japan). Six microscopic fields per mouse were evaluated for inflammation and sinusoidal dilation, which were graded as follows: normal (0), mild (1), moderate (2), and severe (3). If the tissue sections of the liver had a normal tissue structure, a clear and regular lobular arrangement, and regular cell cords and sinusoids had a normal size, and focal and local inflammation was not seen, we gave grade 0. Grade 1 was considered for focal and localized inflammatory cell infiltration to a small degree, and lobular structures were slightly irregular. If these changes were moderate in the liver parenchyma, grade 2 was applied, and if lobular disarray and inflammatory cell infiltration were very high, grade 3 was applied.

### Statistical analysis

Data were statistically assessed utilizing GraphPad Prism software (version 8.0, Inc., La Jolla, CA, USA). One-way ANOVA and Tukey's post hoc test were used for assessing group differences, with p<0.05 considered significant.

## Results

### The effect of syringic acid on liver index

**Figure 1:**
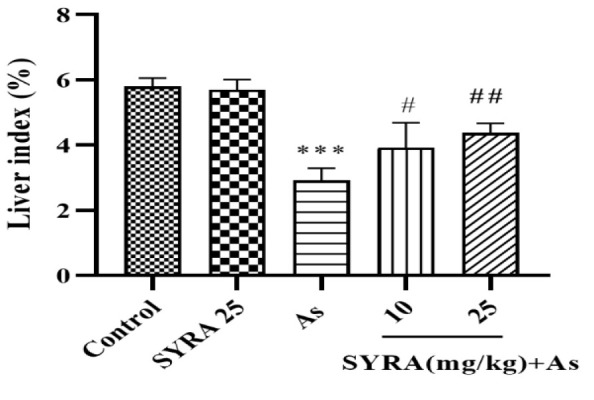
The effect of syringic acid (SYRA) on liver index in sodium arsenite (As) toxicity. *Significantly different from the control (***p<0.001). #significantly different from the As group (#p<0.05 and ##p<0.01).

The effect of SYRA on the liver index (liver weight/body weight) showed that SYRA led to an increase in the liver index of mice exposed to As (Figure 1). 

### The effect of syringic acid on FBS and GTT

**Figure 2:**
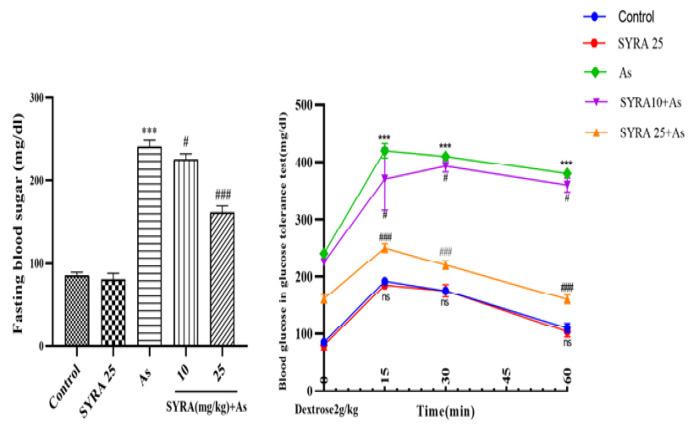
The effect of syringic acid (SYRA) on the levels of fasting blood sugar (FBS) and glucose tolerance test (GTT) in the sodium arsenite (As) exposed mice. *Significantly different from the control (***p<0.001). #Significantly different from the As group (#p<0.05 and ###p<0.001).

After fasting for overnight, FBS and GTT were measured on the 31st day. A significant increase in blood glucose level was observed in As group compared to other groups. SYRA prevented glucose intolerance and hyperglycemia in groups receiving As (Figure 2).

### The effect of syringic acid on liver function markers

Serum levels of ALT, AST, and ALP were significantly elevated in the As group compared to the control group (p<0.001).

**Figure 3:**
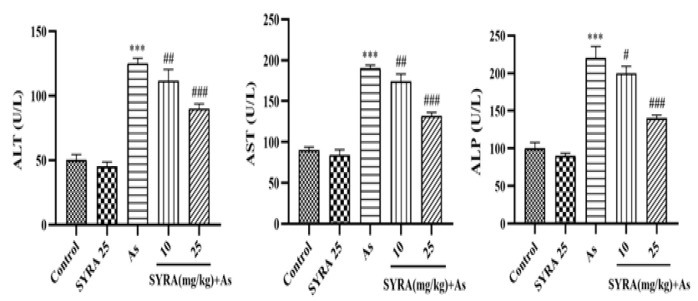
The effect of syringic acid (SYRA) on the activity levels of alanine aminotransferase (ALT), aspartate aminotransferase (AST), and alkaline phosphatase (ALP) in the sodium arsenite (As) intoxicated mice. *Significantly different from the control (***p<0.001). #Significantly different from the As group (#p<0.05, ##p<0.01, and ###p<0.001).

Treatment with SYRA, reduced this increase in a dose-dependent manner. A significant decrease in liver function markers was observed in the group receiving 25 mg/kg SYRA (Figure 3). 

### The effect of syringic acid on oxidative stress biomarkers

**Figure 4:**
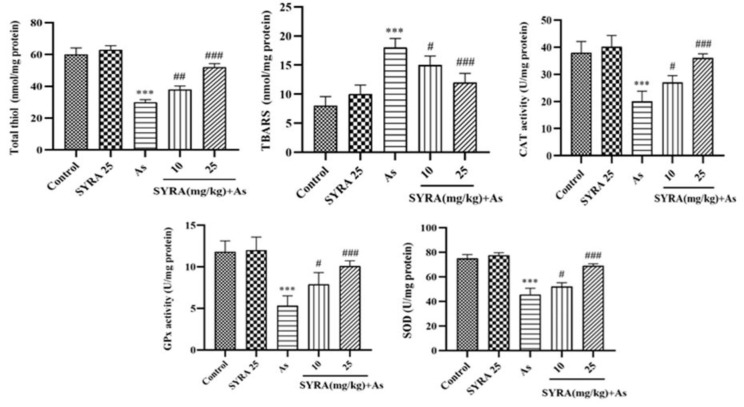
The effect of syringic acid (SYRA) on the hepatic levels of total thiol, TBARS, and activity of CAT, GPx, and SOD in sodium arsenite (As) exposed mice. *Significantly different from the control (***p<0.001). #Significantly different from the As group (#p<0.05, ##p<0.01 and ###p<0.001).

Exposure to As significantly decreased the enzymatic activity of SOD, CAT, and GPx, as well as total thiol content while increasing TBARS levels relative to the control group (p<0.001). SYRA treatment reversed these changes and leading to a significant elevation in antioxidant enzymes activity and total thiol content, and a decrease in TBARS level, particularly at the 25 mg/kg dose (p<0.001) (Figure 4).

### The effect of syringic acid on inflammatory factors

**Figure 5:**
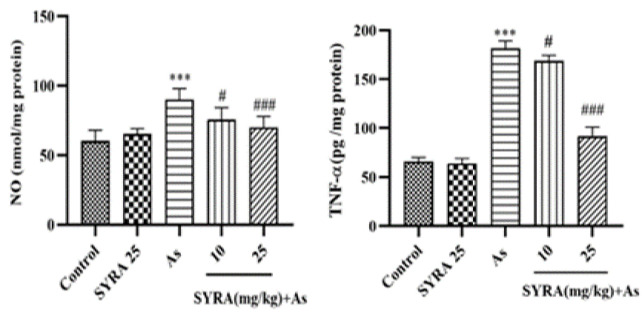
The effect of syringic acid (SYRA) on the hepatic levels of inflammatory biomarkers, nitric oxide (NO) and tumor necrosis factor-alpha (TNF-α) in sodium arsenite (As) intoxicated mice. *Significantly different from the control (***p<0.001). #significantly different from the As group (#p<0.05, and ###p<0.001).

The levels of NO and TNF-α were markedly elevated in the As group compared to the control group (p<0.001). SYRA treatment reduced inflammatory markers, with a substantial effect observed in the group receiving 25 mg/kg SYRA (p<0.001) (Figure 5).

### The effect of syringic acid on the expression of caspase-3 protein

**Figure 6:**
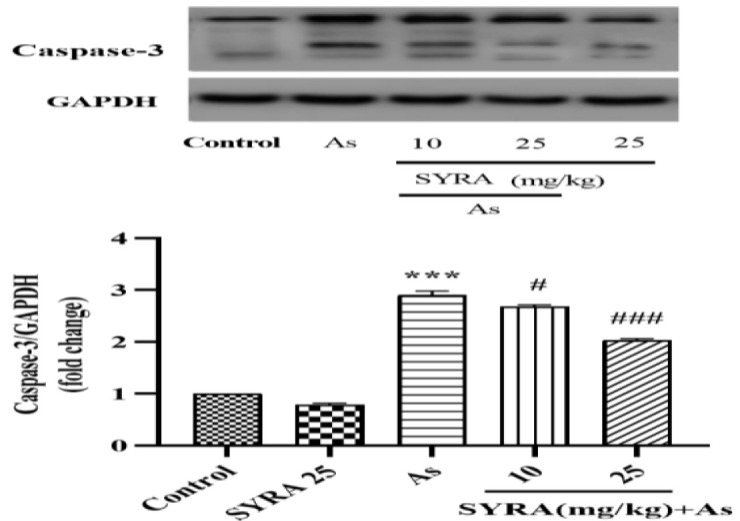
The effect of syringic acid (SYRA) on the hepatic expression of caspase-3 protein in the sodium arsenite (As) intoxicated mice. *Significantly different from the control (***p<0.001). #significantly different from the As group (#p<0.05, and ###p<0.001).

 Caspase-3 protein expression was significantly increased in the As group relative to the control (p<0.001). Co-treatment with SYRA at doses of 10 and 25 mg/kg significantly decreased caspase-3 protein expression (p<0.05 and p<0.001, respectively) compared to the As group (Figure 6).

### The effect of syringic acid on histopathological changes

**Figure 7:**
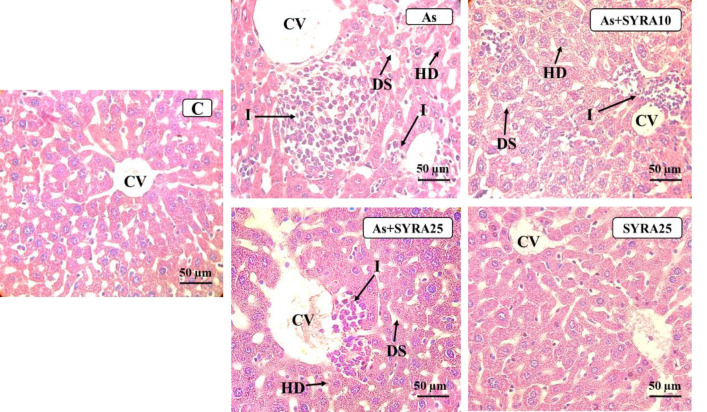
Light microscopy examination of liver tissue stained by H&E in the groups of control (C), sodium arsenite (As), sodium arsenite + syringic acid 10 (As+SYRA 10), sodium arsenite + syringic acid 25 (As+SYRA 25), syringic acid 25 (SYRA 25). Inflammation (I), dilated sinusoids (DS), and disarray of hepatocytes (HD) and lobules were observed in the As group. In the As groups treated with SYRA the widening of the sinusoids and the infiltration of inflammatory cells were reduced compared to the untreated As group. Magnification: ×400.

The liver Hematoxylin and eosin (H&E) staining revealed normal tissue structure, clear and regular lobular arrangement, and a single layer of hepatocytes surrounding the central vein (CV) in the control and SYRA groups. In contrast, the As group exhibited focal and local inflammatory cell infiltration, edema around the central vein, disarray of hepatocytes (HD), and lobules, and dilated sinusoids. These histopathological changes were attenuated in the As+SYRA 10 and As+SYRA 25 groups compared to the As group. The 25 mg/kg dose of SYRA showed a more pronounced protective effect (Figure 7, and Table 1).

**Table 1:** The effect of syringic acid (SYRA) on inflammation and dilation of sinusoids in the sodium arsenite (As) intoxicated mice.

**Dilated sinusoids (%)**	**Inflammation**	**Groups**
0.00 ±0.00	0.11 ±0.04	Control
2.46 ± 0.62***	6.79 ± 1.71***	As
0.00 ±0.00	0.09 ±0.02	SYRA 25
1.85 ± 0.31**^#^	5.14± 1.42***^#^	As + SYRA 10
0.85± 0.07*^##^	2.79 ± 0.71**^##^	As + SYRA 25

Results are presented as the means ± standard deviation. *p<0.05, **p<0.001, and ***p<0.0001 vs the control.  #p<0.05 and ##p<0.001 vs the As group.

## Discussion

This study shows that SYRA reduces liver damage and diabetic conditions induced by As in mice. Consumption of arsenic-contaminated drinking water is a major global health concern affecting millions of people worldwide (Zheng et al., 2013). Epidemiological studies have shown that chronic exposure to arsenic is associated with an increased risk of diabetes and 50% higher mortality. The National Toxicology Program 2011 investigated the relationship between moderate to high exposure to inorganic arsenic (>150 μg/L) in drinking water and diabetes. Taiwan and Bangladesh reported that drinking water with arsenic levels above 100 ppb was associated with a higher risk of diabetes (Root et al., 2023; Sanches et al., 2023). In the present study, chronic exposure to 3 mg/kg arsenic in drinking water resulted in elevated blood glucose level, which is consistent with the study of Singh and Kotikalapudi (Singh et al., 2020, and Kotikalapudi et al., 2023). SYRA is an abundant phenolic compound in plants that has been shown to reduce reactive oxygen species (ROS) and malondialdehyde production. SYRA also ameliorates complications of hepatitis, nephropathy, and neuropathy in diabetic mice, indicating its anti-inflammatory and antidiabetic potential (Sabahi et al., 2021). Therefore, we investigated the efficacy of SYRA in controlling AS-induced glucose intolerance and hepatotoxicity. Our results showed that blood glucose level was reduced in the groups receiving SYRA before As exposure. Histopathological examinations revealed inflammation and dilated sinusoids in the As-exposed group. Meanwhile, administration of SYRA10 and 25 mg/kg resulted in a significant reduction in sinusoidal widening and inflammatory cell infiltration, confirming previous observations (Turk et al., 2019). Arsenic disrupts glucose homeostasis in peripheral tissues and insulin secretion from pancreatic beta cells. As a result, As poisoning leads to decrease in insulin secretion, increase in oxidative stress, increase in gluconeogenesis, and insulin resistance in peripheral tissues (Ahmed et al., 2022; Choi et al., 2009; Rojano-Toimil et al., 2022). Elevated levels of liver enzymes ALT, AST, and ALP are indicative of liver damage, as their release from liver cells into the bloodstream leads to oxidative stress and hepatotoxicity. In our study, As exposure increased liver enzyme activity levels in mice, which is consistent with previous findings (Turk et al., 2019). In addition, decreased antioxidant enzymes activity (SOD, CAT, and GPx) and total thiol level were observed in the As-exposed group, while SYRA-treated groups showed increases in these parameters, which was consistent with the results of Li et al (Li et al., 2020). Previous studies have demonstrated the ability of SYRA to enhance resistance to oxidative stress, reduce serum liver enzyme levels, reduce inflammatory factors, and increase liver antioxidant activity (Cikman et al., 2015; Tan et al., 2023). In this study, co-treatment with As and SYRA 25 mg/kg significantly reduced caspase-3 protein expression compared to the As group. The protective mechanisms of SYRA include enhancing antioxidant defense, reducing lipid peroxidation, inflammation, and regulating caspase-3 protein expression in the liver. The higher dose of SYRA (25 mg/kg) showed significantly more beneficial effects than the lower dose (10 mg/kg).

**Figure 8:**
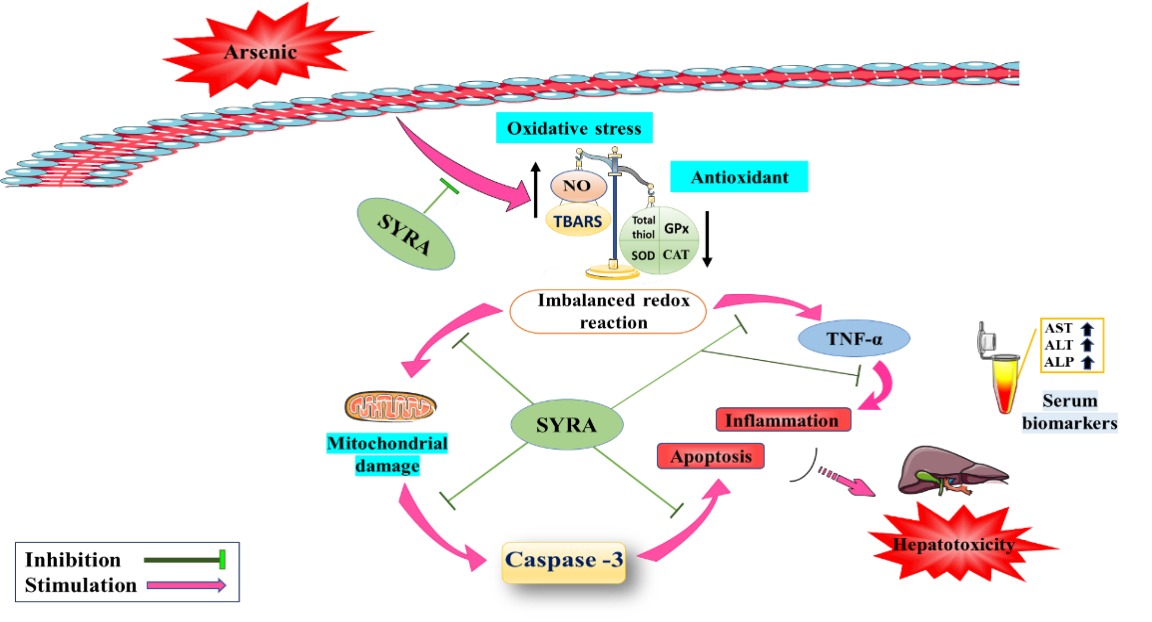
Graphical abstract: The hepatoprotective impacts of syringic acid (SYRA) on arsenic-induced hepatotoxicity. The protective effects of SYRA include reduction in activity of AST, ALT, and ALP, enhancement of cellular antioxidant defense system, reduction of inflammation, oxidative stress, apoptosis, and improvement of As-induced pathological damage in mice.

In summary, this study demonstrates that SYRA attenuates the deleterious effects of As and glucose metabolism in mice liver (Figure 8). The higher dose of SYRA (25 mg/kg) showed significantly more beneficial effects than the lower dose (10 mg/kg). SYRA may have potential therapeutic applications in the prevention and treatment of hepatotoxicity and diabetes. However, further animal studies and clinical trials are necessary to confirm the therapeutic use of SYRA and to investigate its mechanistic pathways in detail.
